# Comparing kinetic profiles between bifunctional and binary type of Zn(salen)-based catalysts for organic carbonate formation

**DOI:** 10.3762/bjoc.10.191

**Published:** 2014-08-08

**Authors:** Carmen Martín, Arjan W Kleij

**Affiliations:** 1 Institute of Chemical Research of Catalonia (ICIQ), Av. Països Catalans 16, 43007 Tarragona, Spain; 2 Catalan Institute for Research and Advanced Studies (ICREA), Pg. Lluis Companys 23, 08010 Barcelona, Spain

**Keywords:** CO_2_ chemistry, cyclic carbonates, kinetic studies, salen complexes, zinc

## Abstract

Zn(salen) complexes have been employed as active catalysts for the formation of cyclic carbonates from epoxides and CO_2_. A series of kinetic experiments was carried out to obtain information about the mechanism for this process catalyzed by these complexes and in particular about the order-dependence in catalyst. A comparative analysis was done between the binary catalyst system Zn(salphen)/NBu_4_I and a bifunctional system Zn(salpyr)·MeI with a built-in nucleophile. The latter system demonstrates an apparent second-order dependence on the bifunctional catalyst concentration and thus follows a different, bimetallic mechanism as opposed to the binary catalyst that is connected with a first-order dependence on the catalyst concentration and a monometallic mechanism.

## Introduction

**Scheme 1:**
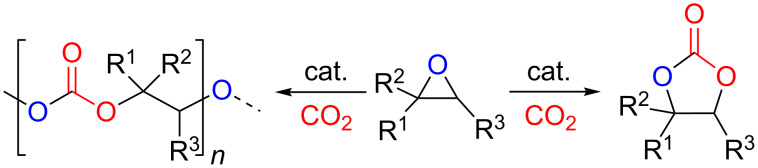
Catalytic synthesis of organic (poly)carbonates from epoxides and CO_2_.

**Figure 1:**
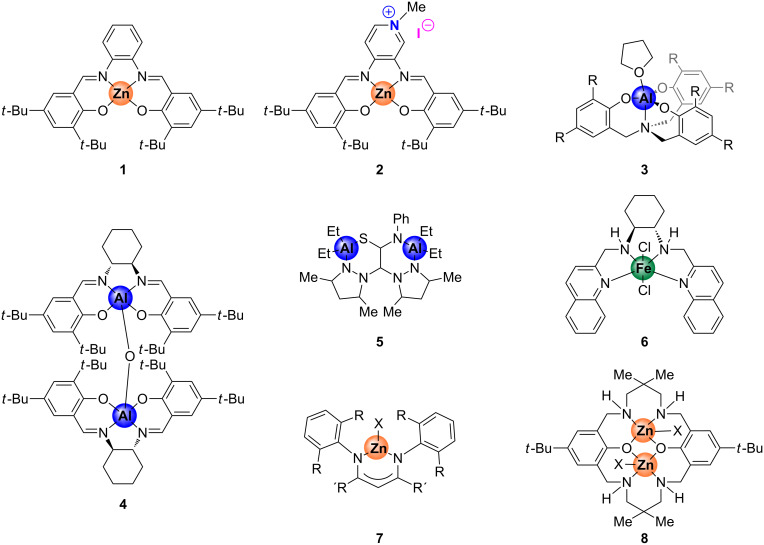
Structures of some metal complexes used as catalyst for (cyclic) organic carbonate synthesis.

**Figure 2:**
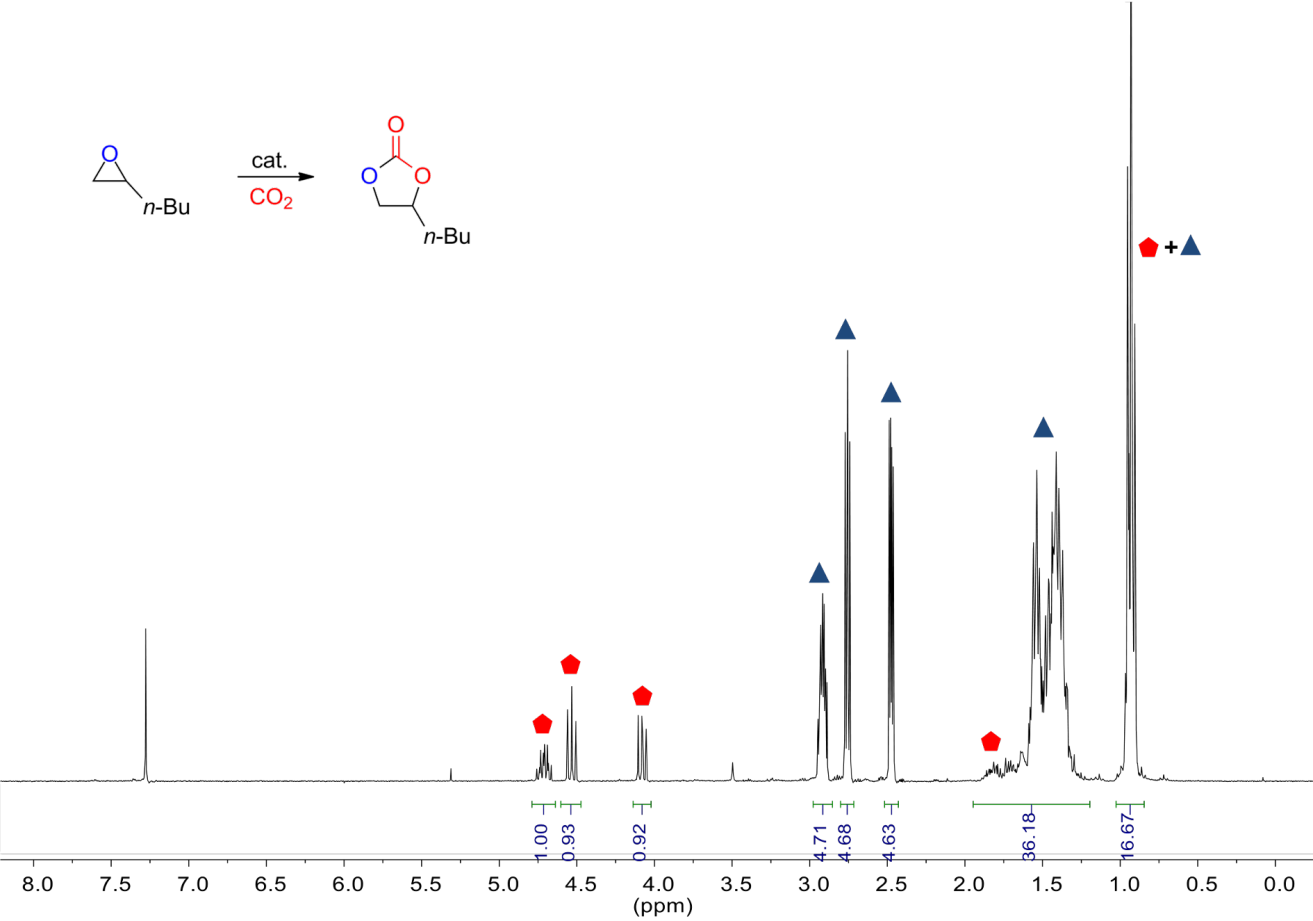
Typical ^1^H NMR spectrum of a sample of a crude mixture in CDCl_3_ (300 MHz) at rt.

Carbon dioxide may be regarded as an ideal, renewable carbon feed stock for the synthesis of organic compounds being also of interest in an industrial context [1–5]. This inexpensive, abundant and nontoxic source of carbon has been extensively used to convert epoxides into their respective cyclic carbonates [6–9] (Scheme 1), that find useful applications as green solvents, precursors towards pharmaceutical intermediates and as electrolytes in lithium ion batteries [1,10–11]. However, energy is required to activate the kinetically highly stable CO_2_ making the use of catalysts a requisite to overcome this limitation and to convert it under more attractive reaction conditions. Over the past years many different catalytic systems have been developed for this kind of process exemplifying the huge interest in the synthesis of cyclic carbonates and these catalysts include quaternary ammonium salts [12], ionic liquids [13–14] and metal-based catalysts [15–21]. In this regard, our group has shown in previous reports various effective organic [22–23] and metal-based systems [24–29] applied as catalysts for organic carbonate formation. Interestingly, we demonstrated high activity and versatility of cheaper, nontoxic and earth-abundant metal-based complexes based on aluminum [24] and iron [25] amino-triphenolate complexes. Additionally, salen-based Zn complexes were also found to be rather efficient catalyst for this transformation. More specifically, these systems relate to the Zn(salphen) family of complexes [salphen = *N*,*N*’-phenylene-1,2-bis[salicylidene]imine] (Figure 1, **1**) combined with a nucleophilic ammonium halide salt [26–27], or an analogous bifunctional system (Figure 1, **2**) containing a Lewis acidic and nucleophilic center within the same molecule [29].

Mechanistic investigations for these CO_2_/epoxide coupling reactions are essential to control the process selectivity (cyclic versus polycarbonate formation, Scheme 1) and to improve the activity of the used catalysts. Several computational investigations have been reported in this area focusing on catalysts comprising ionic liquids [30], *N*-heterocyclic carbenes [31], polyphenolic compounds [32], quaternary ammonium salts [33] or metal-containing complexes [34–37]. Also, a detailed theoretical analysis combined with experimental evidences using a Zn(salphen) complex has been reported [36]. Beside these computational activities, experimental studies involving kinetic measurements have also been undertaken with the aim to obtain more insight into the operating mechanism [15,37–45]. Prominent among these studies is the work carried out by North and co-workers [15,39] who described kinetic studies of a binary, dimetallic aluminum–salen complex **4** (see Figure 1) in conjunction with NBu_4_Br. Remarkably, a second-order dependence on the concentration of the ammonium salt was determined suggesting that two molecules of NBu_4_Br are involved in the rate-determining step: one molecule is supposed to be converted in situ to NBu_3_ able to activate CO_2_ whereas the other molecule helps to ring-open a coordinated epoxide. This activation mode differs from one reported for the binary system based on Al complex **3**/NBu_4_I (Figure 1) [37] and the first-order dependence with respect to each catalyst and co-catalyst displayed when the dimetallic aluminum complex **5** (Figure 1) was utilized [40]. Another interesting example was described by Rieger et al. who used a single-component catalyst **6** based on iron (Figure 1) for which a second-order rate dependence was determined indicating a dimetallic reaction mechanism [41]. Of further importance are the efforts from Coates and co-workers [44] and the kinetic studies described by the group of Williams [45]. In each of these latter cases a binuclear mechanism was proposed for the copolymerization reaction of CO_2_ and epoxide, establishing a second-order dependence for mononuclear Zn complex **7** (Figure 1) employed by Coates and first-order behavior in the case of the dinuclear Zn complex **8** (Figure 1). However, to the best of our knowledge, reports on comparative kinetic studies between structurally related binary and bifunctional systems are rare [43].

Herein we describe such a kinetic investigation of the mechanism of the coupling between CO_2_ and epoxides catalyzed by Zn-based complexes containing salen ligands. A comparative kinetic analysis between the binary system Zn(salphen) **1**/NBu_4_I and bifunctional, alkylated Zn(salpyr) complex **2** [salpyr = *N*,*N′*-bis[salicylidene]-3,4-pyridinediamine] has been carried out and the results are in line with the view that the mechanisms by which the organic carbonate product is formed are essentially different and contrasting to previously reported literature [43].

## Results and Discussion

**Scheme 2:**
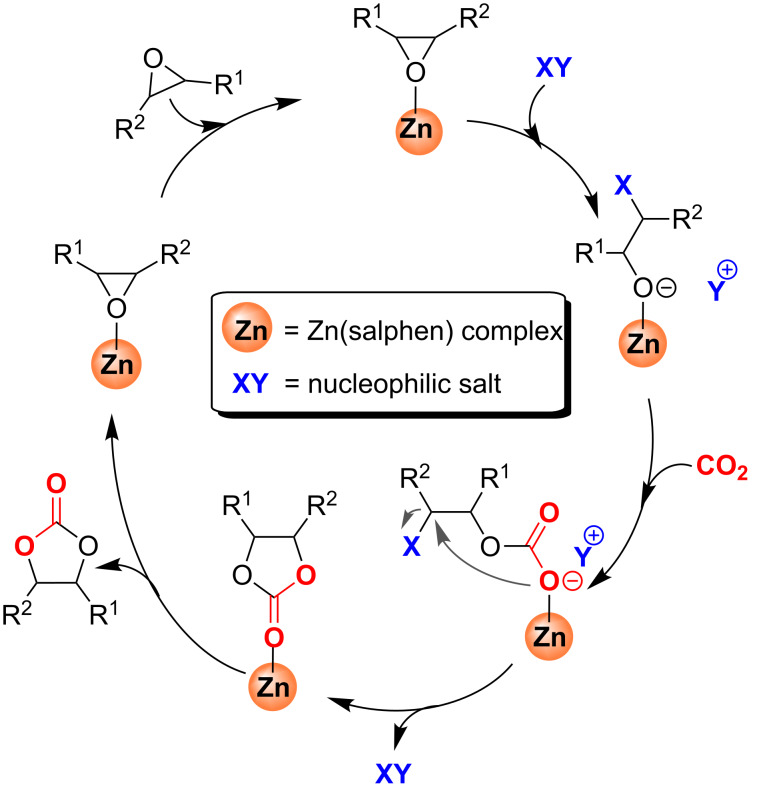
Proposed mechanistic cycle for cyclic carbonate synthesis mediated by Zn(salphen) complexes in the presence of onium salts (XY).

We have described the catalytic capability of Zn(salphen) complexes in conjunction with NBu_4_I for CO_2_/epoxide coupling reactions [26–29], proposing for this process the monometallic mechanism depicted in Scheme 2 [26]. Furthermore, a detailed DFT study for this transformation was reported supporting this proposed mechanism [36]. In the catalytic cycle of Scheme 2 first the epoxide coordinates to the Zn center allowing Lewis acid activation following the ring opening by nucleophilic attack of X. Then, carbon dioxide insertion into the metal–oxygen bond takes place and a consecutive cyclisation step (ring closure) occurs to give the cyclic carbonate and regenerates the (binary) catalyst.

In addition, this transformation has also been developed using a structurally related bifunctional Zn catalyst (Figure 1, **2**) [29] showing a different behavior in terms of reactivity with respect to the binary system. Catalysts based on Zn complexes containing the nucleophile anchored onto the ligand framework are less active than “similar” binary-type catalysts based on comparable components. For instance, coupling between 1,2-epoxyhexane and CO_2_ to afford the cyclic carbonate derivative mediated by binary Zn(salphen) **1**/NBu_4_I was virtually complete whereas the alkylated Zn(salpyr) complex **2** gave a substantially lower 67% result under similar reaction conditions (0.25 mol % of (co)catalyst, 18 h, 1.0 MPa of CO_2_ and 80 °C). On the basis of this difference we decided to investigate the operating mechanisms in more detail through a series of kinetic experiments to obtain more details about the catalytic activation mode of both the binary and bifunctional systems **1**/NBu_4_I and **2** respectively.

In an attempt to assess the role of the catalyst structure in the synthesis of cyclic carbonates, kinetic measurements as a function of catalyst loading were performed (for more details see experimental section). As benchmark substrate 1,2-epoxyhexane was chosen, and in all studied cases complete selectivity towards the cyclic carbonate was observed under neat conditions. The conversion of the substrate was calculated by ^1^H NMR spectroscopy of an aliquot taken from the reaction mixture at the initial stage (at low conversion) of the process assuming “steady-state” conditions working in the presence of an excess of CO_2_ (see Figure 2). Under these reaction conditions the reaction rate can be defined as Equation 1 and further simplified to Equation 2 since both the CO_2_ and epoxide concentration may be considered pseudo constant at the initial stage of the reaction. The natural logarithm of the rate law (Equation 2) results in Equation 3, from which is possible to afford the order “c” with respect to the catalyst concentration by examination of a double logarithmic plot.

[1]
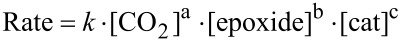


with [cat]^c^ = [Zn]^d^·[I]^e^ in the binary and [cat]^c^ = [ZnI]^c^ in the bifunctional system

[2]
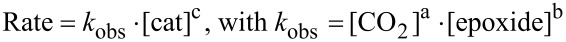


[3]
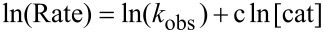


**Scheme 3:**
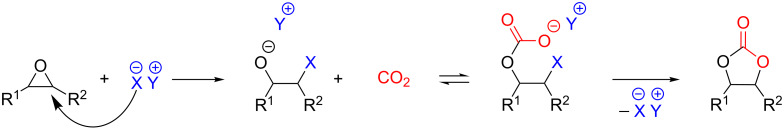
Proposed mechanism for the formation of cyclic carbonates mediated by an ammonium salt.

**Figure 3:**
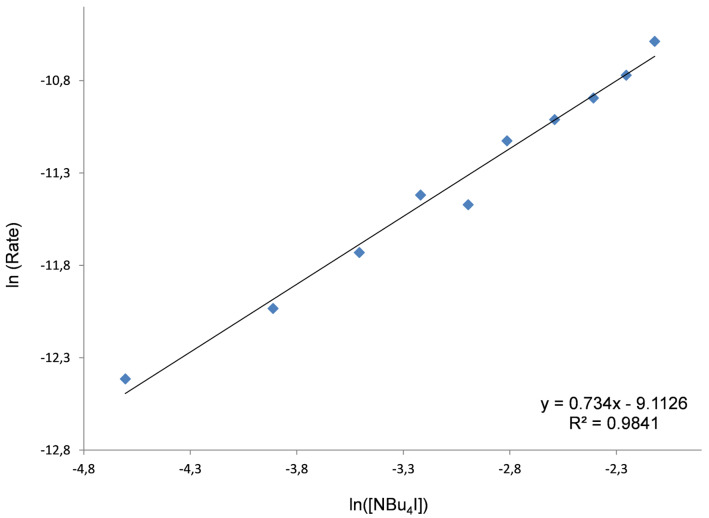
Double logarithmic plot and to determinate the order in NBu_4_I. Conditions: 1,2-epoxyhexane (10 mmol) as substrate, 50 °C, 1.0 MPa of CO_2_, 18 h, [NBu_4_I] = 0.01–0.12 mol %.

Before performing the kinetic experiments with Zn complexes **1** and **2**, the catalytic activity of NBu_4_I (the “co-catalyst”) was first tested where [cat] is equal to [NBu_4_I]. A linear and well-behaved correlation between ln(Rate) and ln([NBu_4_I]) was found (Figure 3) with a gradient of 0.734. This apparent first order in [NBu_4_I] is expected for the synthesis of cyclic carbonates being comparable with that observed for other similar salts [38] with the attack of the nucleophile on the epoxide proposed as the rate-determining step of the mechanism (Scheme 3). This kinetic behavior was evaluated after 18 h at 50 °C in view of the very low catalytic activity of NBu_4_I being virtually absent at ambient temperature. This fact allowed us to analyze the binary system and to study the catalytic activity of the binary couple Zn(salphen) **1**/NBu_4_I since no conversion was observed at 30 °C for NBu_4_I alone after two hours, whereas a significant conversion is achieved by the binary system under these latter conditions.

**Figure 4:**
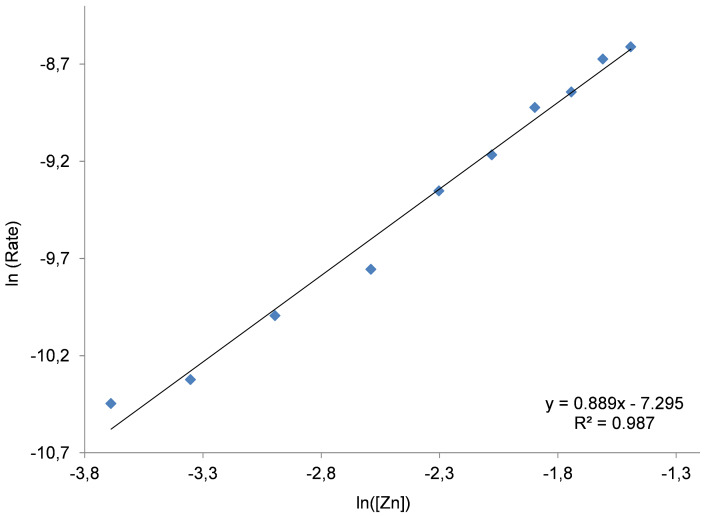
Double logarithmic plot to determine the order in binary catalyst Zn(salphen) **1**/NBu_4_I in the presence of a (small) excess of NBu_4_I. Conditions: 1,2-epoxyhexane (10 mmol) as substrate, 30 °C, 1 MPa of CO_2_, 2 h, [Zn] = 0.025–0.225 mol % and [NBu_4_I] = 0.4 mol %. For the steady state domain using two different concentrations of binary catalyst please refer to Supporting Information File 1, Figure S5.

**Figure 5:**
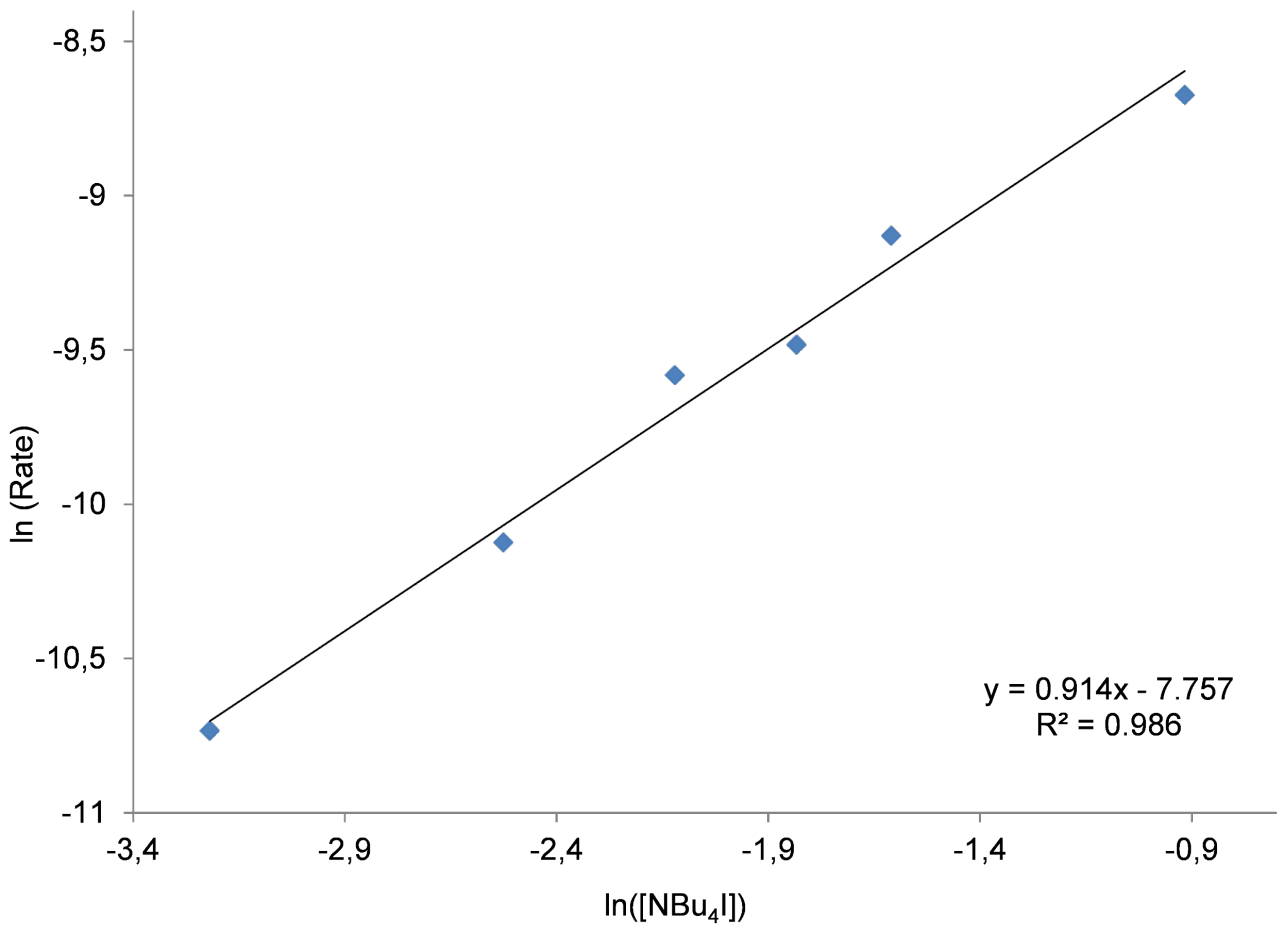
Double logarithmic plot to determine the order in binary catalyst Zn(salphen) **1**/NBu_4_I in the presence of (a small excess of) Zn(salphen) complex **1**. Conditions: 1,2-epoxyhexane (10 mmol) as substrate, 30 °C, 1 MPa of CO_2_, 2 h; [Zn] = 0.2 mol %, [NBu_4_I] = 0.04–0.4 mol %. For the steady state domain using two different concentrations of binary catalyst please refer to Supporting Information File 1, Figure S5.

To evaluate the function of the Zn complex and the NBu_4_I in the binary catalyst system, three different kinetic experiments were carried out using similar reaction conditions (30 °C, 1 MPa of CO_2_, 2 h). Initially, the amount of co-catalyst was fixed at 0.4 mol % whilst the concentration of Zn complex **1** was varied between 0.025–0.225 mol %. In the second series, the concentration of Zn was kept at 0.2 mol %, and altering [NBu_4_I] from 0.04–0.4 mol %. In both cases the double logarithmic plot showed a linear behavior providing a slope of 0.889 and 0.915 respectively (Figure 4 and Figure 5).

The apparent first-order dependence on the binary catalyst system in either case suggests that only one molecule of Zn complex **1** as well as one molecule of ammonium salt is involved in the rate-determining step of the catalytic cycle. Similar results have been reported by Otero and co-workers [40], describing a monometallic mechanism for dimetallic aluminum complex **5** (Figure 1). However, in the literature we can also find different proposals. For instead, North proposed a second-order dependence on NBu_4_Br when using the binary system **4**/NBu_4_Br. A double role of the salt was suggested, providing bromide as nucleophile for the ring opening of the coordinated epoxide and conversely triethylamine which can activate the CO_2_.

**Figure 6:**
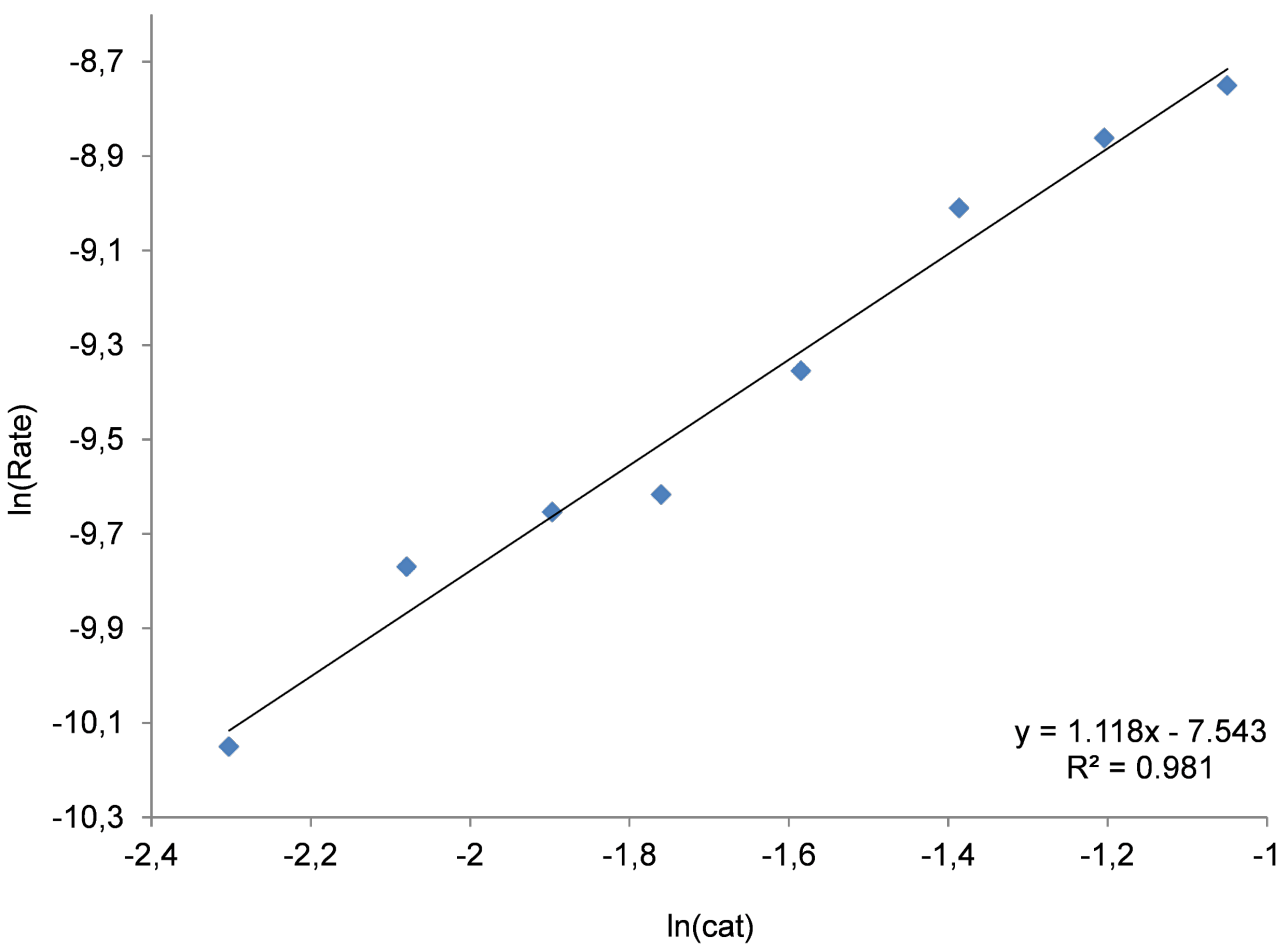
Double logarithmic plot to determine the order with respect to the binary system NBu_4_I/Zn complex **1**. Conditions: 1,2-epoxyhexane (10 mmol) as substrate, 30 °C, 1 MPa of CO_2_, 2 h, [Zn] = [NBu_4_I] = 0.10–0.35 mol %. For the steady state domain using two different concentrations of binary catalyst please refer to Supporting Information File 1, Figure S5.

In addition, we also performed kinetic studies using a fixed ratio (1:1) between the Zn(salphen) complex **1** and NBu_4_I (Figure 6) at different concentrations; also in this case a first-order dependence seems obvious. This result implies that both the Lewis acid and iodide species are important in the rate-determining step, therefore they should not be considered separately. Thus, the first order in catalyst concentration observed in these three different experiments that we performed can be ascribed to the order shown in the catalytic species formed by both the Zn complex **1** and NBu_4_I.

**Figure 7:**
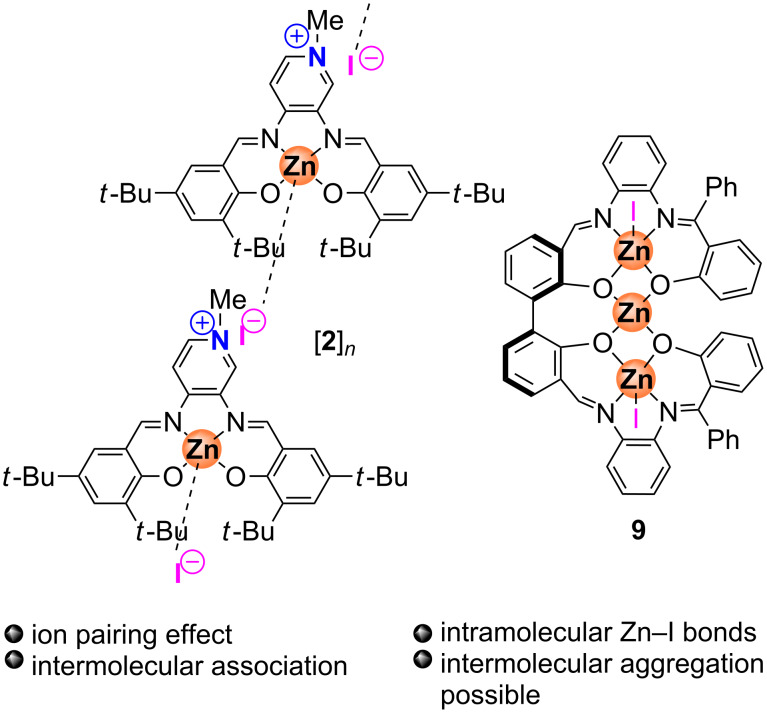
Proposed association for complex **2** and schematic structure for bifunctional complex **9**.

Next we turned our focus on the bifunctional system **2** (structure presented in Figure 1) to examine the order in **2** as to propose a plausible mechanism for the formation of cyclic carbonates mediated by this system. With this in mind kinetic analysis was undertaken by varying the concentration of **2**. It should be noted that in this case an elevated temperature was required to allow for satisfactory product formation. At lower temperatures (rt) complex **2** in solution is likely present in an aggregated form by means of intermolecular interactions (Figure 7) as was recently demonstrated by Santo Di Bella for a similar Zn(salen) complex comprising ammonium bromide functions in the ligand scaffold [46]. The presence of reminiscent Zn–I coordination patterns has also been reported by our group [47]. Another possible barrier to overcome is the (strong) ion-pair effect between the methylpyridinium unit in **2** and the iodide when comparing to the binary system Zn(salphen) **1**/NBu_4_I. Thus, to potentially break the Zn–I coordinative interaction and to enable both Lewis acid and nucleophilic functions, the reaction had to be heated to at least to 40 °C. A similar behavior was observed in previous work from our group for the trinuclear complex **9** (Figure 7) since high temperatures were required for the dissociation process of the coordinating iodides [28].

**Figure 8:**
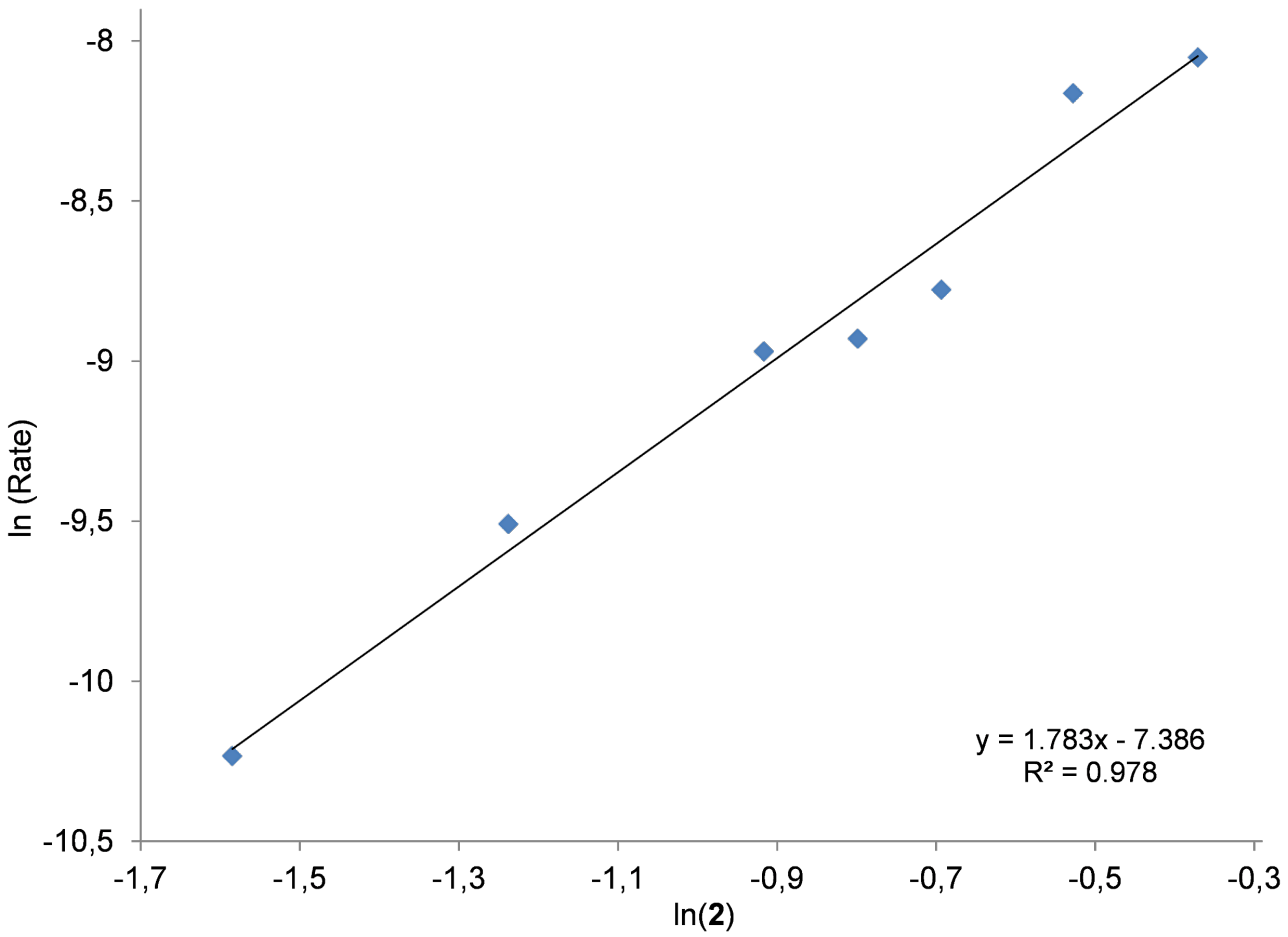
Double logarithmic plot to determine the order in Zn complex **2**. Conditions: 1,2-epoxyhexane (10 mmol) as substrate, 80 °C, 1 MPa of CO_2_, 2 h; [2] = 0.205–0.69 mol %. For the steady state domain using two different concentrations of bifunctional catalyst please refer to Supporting Information File 1, Figure S6.

**Scheme 4:**
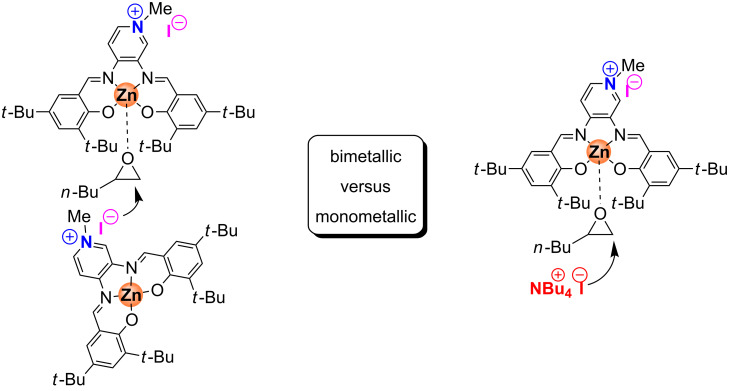
On the left a dimetallic mechanism proposed for bifunctional catalyst **2** and on the right a monometallic mechanism proposed for binary catalyst **2**/NBu_4_I are shown.

Thus, catalytic experiments for bifunctional complex **2** were finally performed at 80 °C and at a CO_2_ pressure of 1 MPa. After 2 h, aliquots of the crude reaction mixtures were checked by ^1^H NMR to determine substrate conversion, and from these data it was possible to correlate the rate with the catalyst concentration in the range 0.205–0.69 mol %. A pseudo-second order was found for the catalytic system indicating a dimetallic reaction mechanism (Figure 8) as was also suggested by Rieger et al. for iron catalyst **6** [41]. This experimental evidence suggests that in the rate-determining step one molecule of the Zn complex **2** may activate the substrate through coordination, while the iodide anion of a second molecule of **2** attacks the coordinated epoxide (Scheme 4).

This mechanistic proposal does not seem to be affected by temperature, since the apparent second-order dependence was also found when the kinetic experiments were executed at 50 °C (see Supporting Information File 1, Figure S2 for more details).

**Figure 9:**
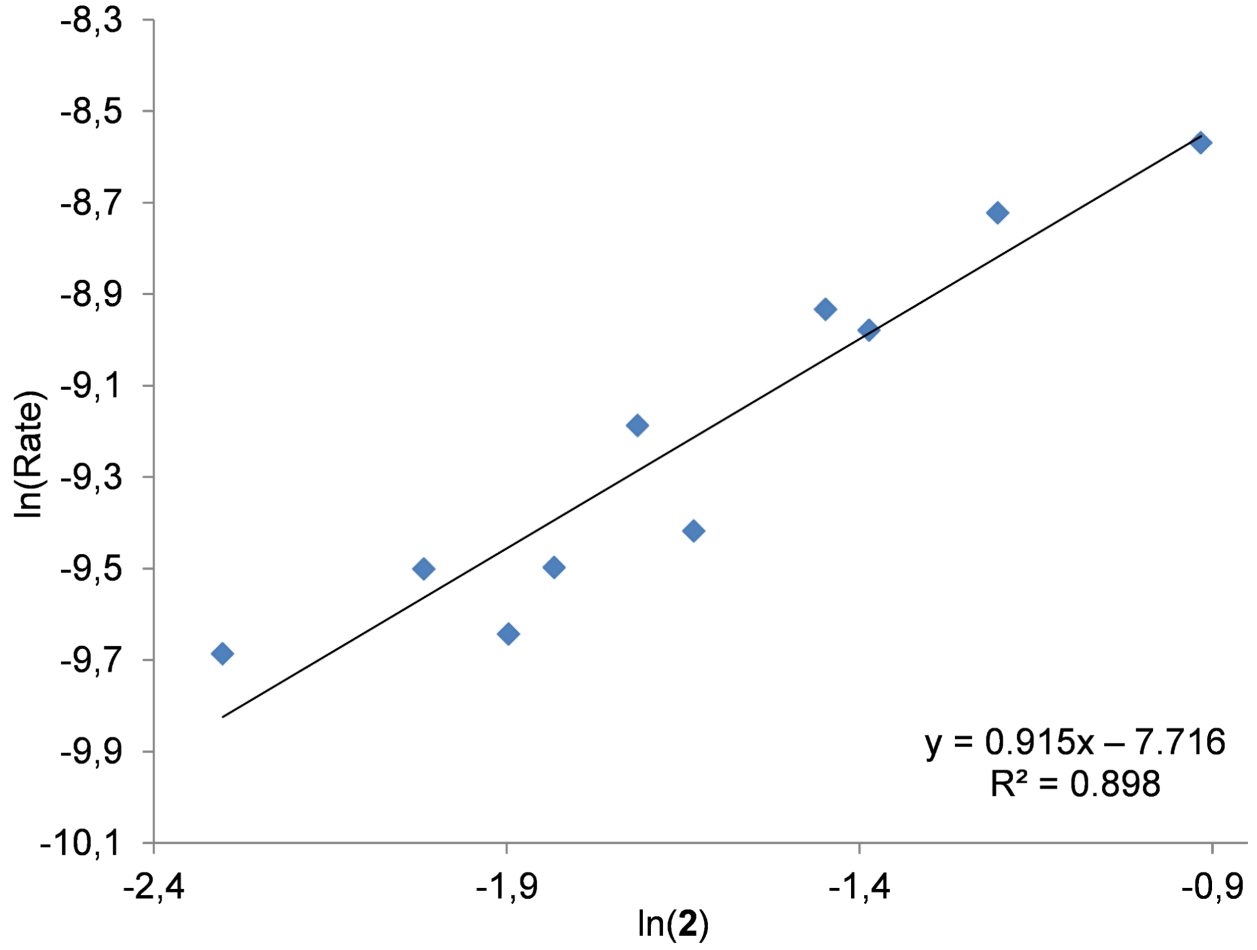
Double logarithmic plot to determine the order with respect to the bifunctional Zn complex **2** in the presence of NBu_4_I. Conditions: 1,2-epoxyhexane (10 mmol) as substrate, 40 °C, 1 MPa of CO_2_, 2 h; [2] = 0.1–0.4 mol %, [NBu_4_I] = 0.6 mol % [48].

To further increase the credibility of a dimetallic mechanism, the bifunctional Zn complex **2** incorporating a methylpyridinium iodide fragment was combined with an external halide source (i.e., NBu_4_I). At relatively low temperature, such a combination should behave (mostly) as a binary system as complex **2** and NBu_4_I alone have lethargic activation behavior. Therefore it may be anticipated that a pseudo first-order dependence results from this combination of catalyst components. We thus performed a series of reactions with a constant NBu_4_I loading (0.6 mol %) while varying the concentration of bifunctional Zn complex **2** (0.1–0.4 mol %). These experiments were carried out at 40 ºC, a minimal reaction temperature required for the bifunctional catalyst to provide a sufficiently disaggregated state and thus the required Lewis acid centers for catalytic turnover when combined with NBu_4_I (Figure 9).

At this temperature individual catalytic conversions for bifunctional complex **2** and ammonium salt alone (2.0 and 2.6%, respectively) are much lower compared with the combination of both (16.5%). Thus, we can assume that the conversion data observed in these experiments correspond mostly with the in situ formed binary catalyst Zn(salpyr)·MeI **2**/NBu_4_I. Under these reaction conditions the initial rate is directly proportional to catalyst concentration and the corresponding double logarithmic plot gives a slope of 0.915 (Figure 9). The effect of the catalyst concentration on the rate for bifunctional complex **2** in the presence or absence of an (external) co-catalyst is markedly different: a pseudo-first order seems apparent for complex **2** in the presence of NBu_4_I consistent with a monometallic mechanism, whereas for bifunctional complex **2** alone the activation of the substrate requires two molecules of **2** (Scheme 4). This is an interesting observation since in the work from Lu et al. [43] an exact opposite observation was done; the comparison between a bifunctional and binary type Co(salen) catalyst for polycarbonate formation revealed that the bifunctional system displays a first order and the binary system a second order rate relationship.

## Conclusion

In summary, kinetic investigations for the addition of CO_2_ into epoxides mediated by Zn(salen) complexes were performed to obtain a more complete understanding of the reaction mechanism. In particular, the binary system **1**/NBu_4_I was compared with the structurally related bifunctional system **2** and both were shown to behave differently. A first-order dependence on catalyst concentration for the binary system was found in line with previous computational work on this system [36] while a second-order rate dependence was observed for the bifunctional catalyst **2**. These observations thus support a monometallic mechanism when Zn species **1** and NBu_4_I are combined, while a bimetallic mechanism seems more likely for the bifunctional complex **2**. The possibility of using this type of alkylammonium halide functionalized ligand in the synthesis of new salpyr-based complexes (M = Co, Cr) allows to reconsider and improve the design of this kind of bifunctional catalyst as to prepare efficient mediators of both cyclic as well as polycarbonates starting from epoxides and CO_2_. In particular the presence of a tethered co-catalyst with a long enough linker between the ligand and the nucleophilic center [49–50] could allow for more efficient combination with the Lewis acid ion thereby creating a higher degree of synergy.

## Experimental

### General procedures

Carbon dioxide was purchased from PRAXAIR and used without further purification. Epoxide substrate and tetrabutylammonium iodide are commercially available and were used as received. Complexes **1** [51] and **2** [27] were prepared according to literature procedures. NMR spectra were recorded using a Bruker AV-300 spectrometer and referenced to the residual NMR solvent signals.

### Typical catalysis procedure

A solution of the respective catalyst in 10 mmol of 1,2-epoxyhexane (1.2 mL, 1.0 g) was transferred to a stainless steel reactor (30 mL). Three cycles of pressurization and depressurization of the reactor with carbon dioxide (0.5 MPa) were carried out to replace all air by CO_2_ in the reactor. The final pressure was then adjusted to 1.0 MPa, and the reaction was left stirring at the required temperature for 2 or 18 h, depending on the experiment. Afterwards, the conversion was calculated by ^1^H NMR spectroscopy of an aliquot of reaction mixture using CDCl_3_ as solvent. Reactions carried out in the presence or absence of mesitylene as an internal standard gave similar results (see Supporting Information File 1, Table S8 for more details). The carbonate product, 4-butyl-1,3-dioxolan-2-one, has been previously described, therefore its identification was done by comparison with reported data [52]. For a photograph of the reactor see Supporting Information File 1.

### Kinetic experiments

For the catalytic studies, the same procedure was used but doing the reaction in an SPR16 Slurry Phase Reactor (Amtec GmbH). The AMTEC vessels were charged with the corresponding catalyst. First a leak test was performed with 1.5 MPa of N_2_ to finally reduce the pressure to 0.2 MPa. Then, the reactors were subjected to three cycles of pressurization and depressurization with CO_2_ (from 0.4 to 0.2 MPa). Finally 10 mmol of 1,2-epoxyhexane was injected into the reactors and the CO_2_ pressure was raised to 1 MPa. Reactions were stirred at the appropriate temperature for two hours. At the end of the process stirring was stopped, reactors were cooled and depressurized. The conversion of the substrate was examined by ^1^H NMR spectroscopy (CDCl_3_) of an aliquot taken from the crude reaction mixture. For a photograph of the Amtec reactor system see Supporting Information File 1.

## Supporting Information

Further details of experimental procedures, typical ^1^H NMR spectra for the aliquots taken in the kinetic studies (containing the carbonate product [4-butyl-1,3-dioxolan-2-one]) and photographs of the reactor systems used for the catalytic/kinetic studies are given.

File 1 Experimental procedures, typical ^1^H NMR spectra for aliquots taken in the kinetic studies and photographs of the reactor systems.

## References

[1] Sakakura T, Choi J-C, Yasuda H (2007). Chem Rev.

[2] Aresta M (2010). Carbon Dioxide as Chemical Feedstock.

[3] Martín R, Kleij A W (2011). ChemSusChem.

[4] Kleij A W (2013). ChemCatChem.

[5] Maeda C, Miyazaki Y, Ema T (2014). Catal Sci Technol.

[6] Decortes A, Castilla A M, Kleij A W (2010). Angew Chem, Int Ed.

[7] North M, Pasquale R, Young C (2010). Green Chem.

[8] Pescarmona P P, Taherimehr M (2012). Catal Sci Technol.

[9] Whiteoak C J, Kleij A W (2013). Synlett.

[10] Schäffner B, Schäffner F, Verevkin S P, Börner A (2010). Chem Rev.

[11] Shaikh A-A G, Sivaram S (1996). Chem Rev.

[12] Caló V, Nacci A, Monopoli A, Fanizzi A (2002). Org Lett.

[13] Yang Z-Z, He L-N, Miao C-X, Chanfreau S (2010). Adv Synth Catal.

[14] Zhao Y, Yao C, Chen G, Yuan Q (2013). Green Chem.

[15] Clegg W, Harrington R W, North M, Pasquale R (2010). Chem–Eur J.

[16] Ren W-M, Wu G-P, Lin F, Jiang J-Y, Liu C, Luo Y, Lu X-B (2012). Chem Sci.

[17] Buchard A, Kember M R, Sandeman K G, Williams C K (2011). Chem Commun.

[18] Chang T, Jin L, Jing H (2009). ChemCatChem.

[19] North M, Villuendas P, Young C (2009). Chem–Eur J.

[20] Paddock R L, Nguyen S T (2004). Chem Commun.

[21] Langanke J, Greiner L, Leitner W (2013). Green Chem.

[22] Whiteoak C J, Henseler A H, Ayats C, Kleij A W, Pericàs M A (2014). Green Chem.

[23] Whiteoak C J, Nova A, Maseras F, Kleij A W (2012). ChemSusChem.

[24] Whiteoak C J, Kielland N, Laserna V, Escudero-Adán E C, Martin E, Kleij A W (2013). J Am Chem Soc.

[25] Whiteoak C J, Martin E, Martinez-Belmonte M, Benet-Buchholz J, Kleij A W (2012). Adv Synth Catal.

[26] Decortes A, Martinez-Belmonte M, Benet-Buchholz J, Kleij A W (2010). Chem Commun.

[27] Decortes A, Kleij A W (2011). ChemCatChem.

[28] Escárcega-Bobadilla M V, Martinez-Belmonte M, Martin E, Escudero-Adán E C, Kleij A W (2013). Chem–Eur J.

[29] Martin C, Whiteoak C J, Martin E, Martinez-Belmonte M, Escudero-Adán E C, Kleij A W (2014). Catal Sci Technol.

[30] Sun H, Zhang D (2007). J Phys Chem A.

[31] Ajitha M J, Suresh C H (2011). Tetrahedron Lett.

[32] Wang J-Q, Sun J, Cheng W-G, Dong K, Zhang X-P, Zhang S-J (2012). Phys Chem Chem Phys.

[33] Wang J-Q, Dong K, Cheng W-G, Sun J, Zhang S-J (2012). Catal Sci Technol.

[34] Man M L, Lam K C, Sit W N, Ng S M, Zhou Z, Lin Z, Lau C P (2006). Chem–Eur J.

[35] Adhikari D, Nguyen S T, Baik M-H (2014). Chem Commun.

[36] Castro-Gómez F, Salassa G, Kleij W A, Bo C (2013). Chem–Eur J.

[37] Whiteoak C J, Kielland N, Laserna V, Castro-Gómez F, Martin E, Escudero-Adán E C, Bo C, Kleij W A (2014). Chem–Eur J.

[38] Kihara N, Hara N, Endo T (1993). J Org Chem.

[39] North M, Pasquale R (2009). Angew Chem, Int Ed.

[40] Castro-Osma J A, Lara-Sánchez A, North M, Otero A, Villuendas P (2012). Catal Sci Technol.

[41] Dengler J-E, Lehenmeier M W, Klaus S, Anderson C E, Herdtweck H, Rieger B (2011). Eur J Inorg Chem.

[42] Darensbourg D J, Yarbrough J C, Ortiz C, Fang C C (2003). J Am Chem Soc.

[43] Liu J, Ren W-M, Liu Y, Lu X-B (2013). Macromolecules.

[44] Moore D R, Cheng M, Lobkovsky E B, Coates G W (2003). J Am Chem Soc.

[45] Jutz F, Buchard A, Kember M R, Fredriksen S B, Williams C K (2011). J Am Chem Soc.

[46] Oliveri I P, Failla S, Colombo A, Dragonetti C, Righetto S, Di Bella S (2014). Dalton Trans.

[47] Wezenberg S J, Escudero-Adán E C, Benet-Buchholz J, Kleij A W (2009). Chem–Eur J.

[R48] 48The scattering in the data is probably a result of the background reactions that are mediated by the bifunctional catalyst **2** and NBu_4_I alone compared with the combination of both. Note that both higher as well as lower reaction temperatures are not feasible due to the absence of sufficient activity (lower temperatures) and too much interference with background activity by both **2** and NBu_4_I at higher reaction temperatures.

[49] Zhang X, Jia Y-B, Lu X-B, Li B, Wang H, Sun L-C (2008). Tetrahedron Lett.

[50] Ren W-M, Liu Z-W, Wen Y-Q, Zhang R, Lu X-B (2009). J Am Chem Soc.

[51] Kleij A W, Kuil M, Tooke D M, Lutz M, Spek A L, Reek J N H (2005). Chem–Eur J.

[52] Jiang J-L, Gao F, Hua R, Qiu X (2005). J Org Chem.

