# Supplementary material for: Comparing kinetic profiles between bifunctional and binary type of Zn(salen)-based catalysts for organic carbonate formation
Source: Beilstein J Org Chem. 2014 Aug 8;10:1817–25. doi: 10.3762/bjoc.10.191 (PMC4143085; doi:10.3762/bjoc.10.191)
Supplement: File 1 — Experimental procedures, typical 1H NMR spectra for aliquots taken in the kinetic studies and photographs of the reactor systems. [file Beilstein_J_Org_Chem-10-1817-s001.pdf]

Supporting Information

for

Comparing kinetic profiles between bifunctional and binary type of Zn(salen)-based catalysts for organic carbonate formation

Carmen Martín[1] and Arjan W. Kleij[*1,2]

Address: [1]Institute of Chemical Research of Catalonia (ICIQ), Av. Països Catalans 16, 43007 Tarragona, Spain and [2]Catalan Institute for Research and Advanced Studies (ICREA), Pg. Lluis Companys 23, 08010 Barcelona, Spain

Email: Arjan W. Kleij - akleij@iciq.es

*Corresponding author

Contents

Typical ^{1}H NMR spectrum of a product of the kinetic experiments	S2
Spectroscopic data of epoxide and cyclic carbonate product	S3
Additional kinetic data	S4
Tables containing conversions and rates of kinetic experiments	S5
Photographs of the reactors used for the catalytic studies	S12
Comparison of reactions carried out in the absence/presence of an internal standard	S13
Determination of steady-state domains:	S14

Typical ^{1}H NMR spectrum of a product of the kinetic experiments:

Figure S1. ^{1}H NMR spectrum of a crude mixture in $CDCl_3$ (300 MHz) at RT.

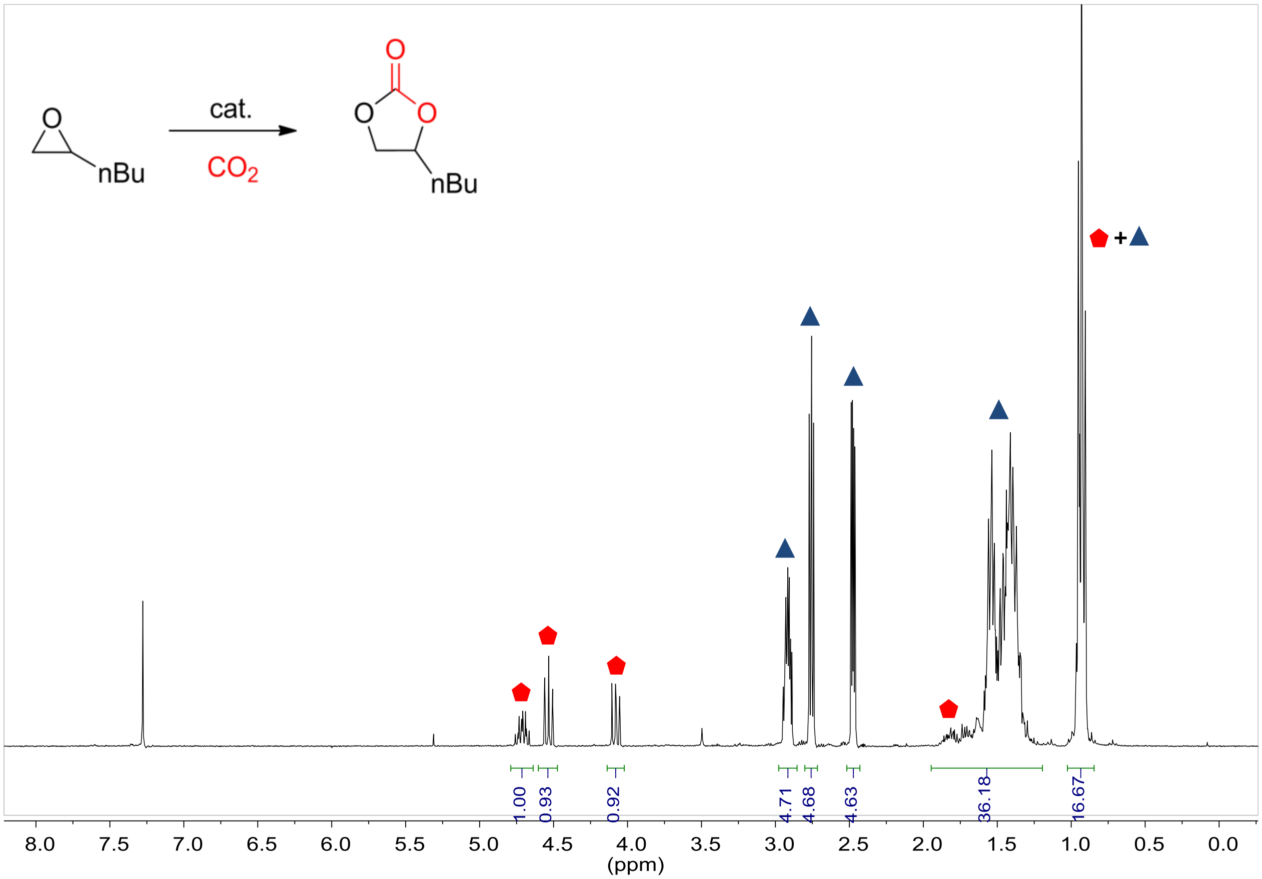

The red pentagons represent the cyclic carbonate product whereas the blue triangles are related to the starting epoxide (1,2-epoxyhexane).

Spectroscopic data of epoxide and cyclic carbonate product:

1,2-Epoxybutane [1] (blue trace in Figure S1)

^{1}H NMR (300 MHz, $CDCl_3$): δ 2.95 – 2.88 (m, 1H), 2.76 (dd, $^2J_{HH}$ = 5.1, $^3J_{HH}$ = 4.0 Hz, 1H), 2.47 (dd, $^2J_{HH}$ = 5.1, $^3J_{HH}$ = 2.7 Hz, 1H), 1.60 – 1.30 (m, 6H), 0.93 (t, $^3J_{HH}$ = 7.0 Hz, 3H).

4-butyl-1,3-dioxolan-2-one [2] (red trace in Figure S1)

^{1}H NMR (300 MHz, $CDCl_3$): δ 4.76 – 4.66 (m, 1H), 4.53 (dd, $^2J_{HH}$ = 8.2, $^3J_{HH}$ = 8.0 Hz, 1H), 4.08 (dd, $^2J_{HH}$ = 8.3, $^3J_{HH}$ = 7.3 Hz, 1H), 1.90 – 1.61 (m, 2H), 1.53 – 1.30 (m, 4H), 0.94 (t, $^3J_{HH}$ = 7.1 Hz, 3H).

---

[1] Substrate purchased from Sigma Aldrich.

[2] Jiang, J.-L.; Gao, F.; Hua, R.; Qiu, X.; *J. Org. Chem.*, 2005, **70**, 381 – 383. doi: 10.1021/jo0485785

Additional kinetic data:

Figure S2: Double logarithmic plot to determinate the order with respect to the bifunctional Zn-complex **2** at 50ºC, 1 MPa CO_2 pressure, 18 h and using 1,2-epoxyhexane as substrate.

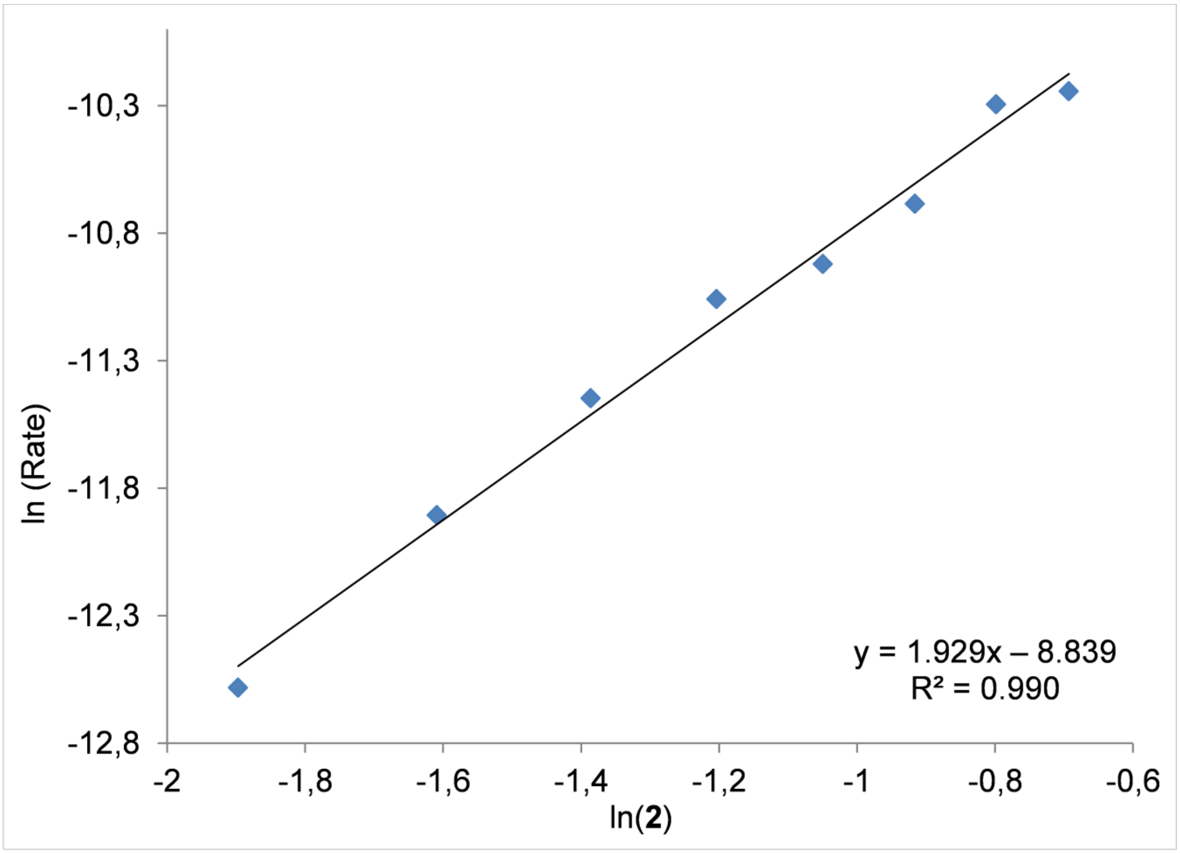

Tables containing conversions and rates of kinetic experiments:

Table S1. Effect of NBu_4I loading on the rate.[a]

[cat]	conv %[b]	Conv mol·L^{-1}	Rate mol·L^{-1} S^{-1}	Ln(Rate)	Ln [cat]
0.12	19.7	1.6351	2.5233E-05	-10.5874	-2.12026
0.105	16.4	1.3612	2.10062E-05	-10.7707	-2.25379
0.09	14.5	1.2035	1.85725E-05	-10.8938	-2.40795
0.075	12.9	1.0707	1.65231E-05	-11.0107	-2.59027
0.06	11.5	0.9545	1.47299E-05	-11.1256	-2.81341
0.05	8.14	0.67562	1.04262E-05	-11.4712	-2.99573
0.04	8.57	0.71131	1.0977E-05	-11.4197	-3.21888
0.03	6.28	0.52124	8.04383E-06	-11.7306	-3.50656
0.02	4.64	0.38512	5.94321E-06	-12.0333	-3.91202
0.01	3.17	0.26311	4.06034E-06	-12.4142	-4.60517

[a] General conditions: 1,2-epoxyhexane (10 mmol), catalyst (0.12 – 0.01 mol%), 18 h, $p(CO_2)$ = 1.0 MPa, 50°C, neat. [b] Conversion determined by ^{1}H NMR ($CDCl_3$); selectivity for the cyclic carbonate, >99%.

Table S2. Effect of the loading of **1** on the rate in the presence of an excess of NBu_4I.[a]

[cat]	conv %[b]	Conv mol·L^{-1}	Rate mol·L^{-1}·S^{-1}	Ln(Rate)	Ln [cat]
0.225	15.79	1.31057	0.000182024	-8.61137	-1.49165
0.2	14.83	1.23089	0.000170957	-8.6741	-1.60944
0.175	12.52	1.03916	0.000144328	-8.84342	-1.74297
0.15	11.56	0.95948	0.000133261	-8.9232	-1.89712
0.125	9.06	0.75198	0.000104442	-9.16688	-2.07944
0.1	7.53	0.62499	8.68042E-05	-9.35186	-2.30259
0.075	5.03	0.41749	5.79847E-05	-9.75533	-2.59027
0.05	3.96	0.32868	0.00004565	-9.99451	-2.99573
0.035	2.85	0.23655	3.28542E-05	-10.3234	-3.35241
0.025	2.52	0.20916	0.00002905	-10.4465	-3.68888

[a] General conditions: 1,2-epoxyhexane (10 mmol), catalyst **1** (0.225 – 0.025 mol%), co-catalyst (0.4 mol%), 2 h, $p(CO_2)$ = 1.0 MPa, 30°C, neat. [b] Conversion determined by ^{1}H NMR ($CDCl_3$); selectivity for the cyclic carbonate, >99%.

Table S3. Effect of the loading of NBu_4I on the rate in the presence of an excess of **1**.[a]

[cat]	conv %[b]	Conv mol·L^{-1}	Rate mol L^{-1}·S^{-1}	Ln (Rate)	Ln [cat]
0.4	14.83	1.23089	0.000170957	-8.6741	-0.91629
0.2	9.4	0.7802	0.000108361	-9.13004	-1.60944
0.16	6.6	0.5478	7.60833E-05	-9.48368	-1.83258
0.12	5.98	0.49634	6.89361E-05	-9.58233	-2.12026
0.08	3.48	0.28884	4.01167E-05	-10.1237	-2.52573
0.04	1.89	0.15687	2.17875E-05	-10.7342	-3.21888

[a] General conditions: 1,2-epoxyhexane (10 mmol), catalyst Zn **1** (0.20 mol%), co-catalyst (0.40 – 0.04 mol%), 2 h, $p(CO_2)$ = 1.0 MPa, 30°C, neat. [b] Conversion determined by ^1H NMR ($CDCl_3$); selectivity for the cyclic carbonate, >99%.

Table S4. Effect of the loading of **1** and NBu_4I on the rate.[a]

[cat]	conv %[b]	Conv mol·L^{-1}	Rate mol·L^{-1}·S^{-1}	Ln(Rate)	Ln [cat]
0.35	13.74	1.14042	0.000158392	-8.75044	-1.04982
0.3	12.3	1.0209	0.000141792	-8.86115	-1.20397
0.25	10.6	0.8798	0.000122194	-9.0099	-1.38629
0.205	7.51	0.62333	8.65736E-05	-9.35452	-1.58475
0.172	5.78	0.47974	6.66306E-05	-9.61635	-1.76026
0.15	5.57	0.46231	6.42097E-05	-9.65336	-1.89712
0.125	4.96	0.41168	5.71778E-05	-9.76935	-2.07944
0.1	3.39	0.28137	3.90792E-05	-10.1499	-2.30259

[a] General conditions: 1,2-epoxyhexane (10 mmol), catalyst **1**/NBu_4I (0.35 – 0.10 mol%), 2 h, $p(CO_2)$ = 1.0 MPa, 30°C, neat. [b] Conversion determined by ^1H NMR ($CDCl_3$); selectivity for the cyclic carbonate, >99%.

Table S5. Effect of the loading of **2** on the rate at 80º C.[a]

[cat]	conv %[b]	Conv mol·L^{-1}	Rate mol·L^{-1}·S^{-1}	Ln(Rate)	Ln [cat]
0.69	27.65	2.29495	0.000318743	-8.05113	-0.37106
0.59	24.72	2.05176	0.000284967	-8.16314	-0.52763
0.5	13.38	1.11054	0.000154242	-8.77699	-0.69315
0.45	11.48	0.95284	0.000132339	-8.93014	-0.79851
0.4	11.04	0.91632	0.000127267	-8.96923	-0.91629
0.29	6.44	0.53452	7.42389E-05	-9.50822	-1.23787
0.205	3.12	0.25896	3.59667E-05	-10.2329	-1.58475

[a] General conditions: 1,2-epoxyhexane (10 mmol), catalyst **2** (0.69 – 0.205 mol%), 2 h, $p(CO_2)$ = 1.0 MPa, 80°C, neat. [b] Conversion determined by ^{1}H NMR ($CDCl_3$); selectivity for the cyclic carbonate, >99%.

Table S6. Effects of the loading of **2** on the rate at 50º C.[a]

[cat]	conv %[b]	Conv mol·L^{-1}	Rate mol·L^{-1}·S^{-1}	Ln(Rate)	Ln [cat]
0.5	27.8	2.3074	3.5608E-05	-10.2429	-0.69315
0.45	26.4	2.1912	3.38148E-05	-10.2946	-0.79851
0.4	17.88	1.48404	2.29019E-05	-10.6843	-0.91629
0.35	14.11	1.17113	1.8073E-05	-10.9211	-1.04982
0.3	12.3	1.0209	1.57546E-05	-11.0584	-1.20397
0.25	8.34	0.69222	1.06824E-05	-11.4469	-1.38629
0.2	5.27	0.43741	6.75015E-06	-11.9059	-1.60944
0.15	2.68	0.22244	3.43272E-06	-12.5822	-1.89712

[a] General conditions: 1,2-epoxyhexane (10 mmol), catalyst **2** (0.50 – 0.15 mol%), 18 h, $p(CO_2)$ = 1.0 MPa, 50°C, neat. [b] Conversion determined by ^{1}H NMR ($CDCl_3$); selectivity for the cyclic carbonate, >99%.

Table S7. Effect of the loading of **2** on the rate in the presence of an excess of NBu_4I.[a.]

[cat]	conv %[b]	Conv mol·L^{-1}	Rate mol·L^{-1}·S^{-1}	Ln(Rate)	Ln [cat]
0.4	16.48	1.36784	0.000189978	-8.5686	-0.91629
0.3	14.13	1.17279	0.000162888	-8.72245	-1.20397
0.25	10.94	0.90802	0.000126114	-8.97833	-1.38629
0.235	11.45	0.95035	0.000131993	-8.93276	-1.44817
0.195	7.05	0.58515	8.12708E-05	-9.41772	-1.63476
0.18	8.88	0.73704	0.000102367	-9.18695	-1.7148
0.16	6.51	0.54033	7.50458E-05	-9.49741	-1.83258
0.15	5.63	0.46729	6.49014E-05	-9.64264	-1.89712
0.133	6.49	0.53867	7.48153E-05	-9.50049	-2.01741
0.1	5.39	0.44737	6.21347E-05	-9.68621	-2.30259

[a] General conditions: 1,2-epoxyhexane (10 mmol), catalyst **2** (0.40 – 0.10 mol%), co-catalyst (0.6 mol%), 2 h, $p(CO_2)$ = 1.0 MPa, 40°C, neat. [b] Conversion determined by ^{1}H NMR ($CDCl_3$); selectivity for the cyclic carbonate, >99%.

Photographs of the reactors used for the catalytic studies:

Figure S3. Standard autoclaves used for the kinetic experiments mentioned in the table S4 and S5.

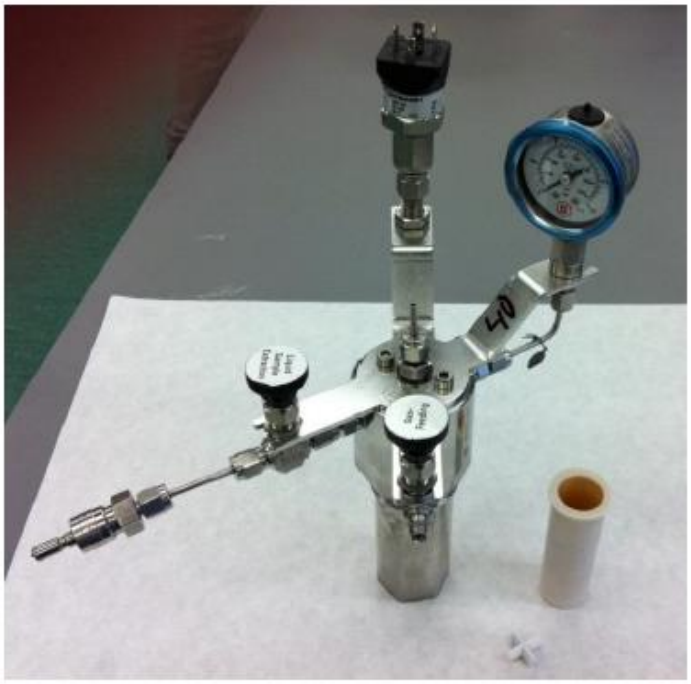

Figure S4. AMTEC SPR-16 system used for the kinetic studies.

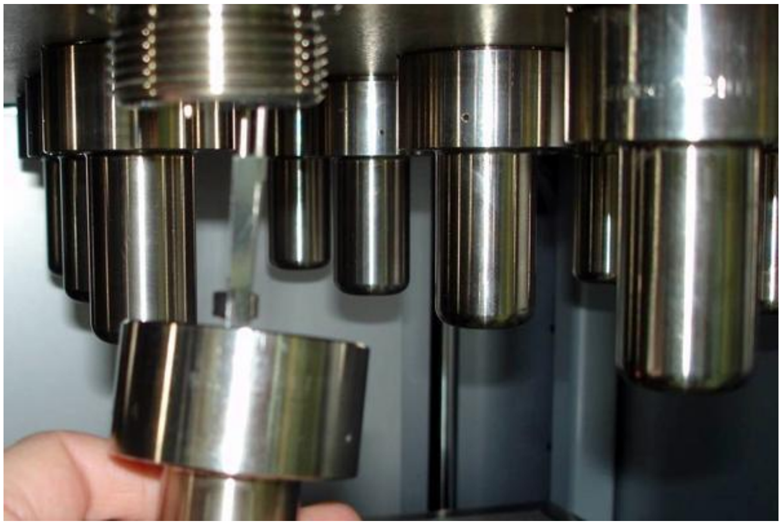

Comparison of reactions carried out in the absence/presence of an internal standard:

- To quantify the amount of carbonate formed, mesitylene was used as internal standard (IS) in several cases. The deviation found was minimal when the amount of carbonate was calculated in the presence or absence of the internal standard. The benchmark substrate (1,2-epoxyhexane) was chosen for this comparative study complete selectivity towards the cyclic carbonate was noted in all studied cases. Thus it is possible to obtain the conversion by integration of the peaks related to the presence of cyclic carbonate formed and the unreacted epoxide with minimal error (see table below).

Table S8:

Catalyst	% cyclic carbonate in absence of IS	% cyclic carbonate in presence of IS
0.69 mol % cat. **2**	27.65	27.66
0.45 mol % cat. **2**	11.48	11.61
0.29 mol % cat. **2**	6.44	6.23
0.25 mol % cat. **2**	7.51	4.92
0.60 mol % cat. **1**	25.90	22.62

Conditions: 1,2-epoxyhexane (10 mmol) as substrate, 1 MPa of CO_2, 2 h, 80 ºC with catalyst **2** and 30ºC with catalyst **1**.

Determination of steady-state domains:

Figure S5:

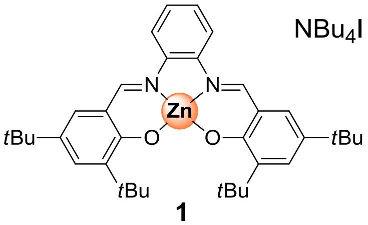

1

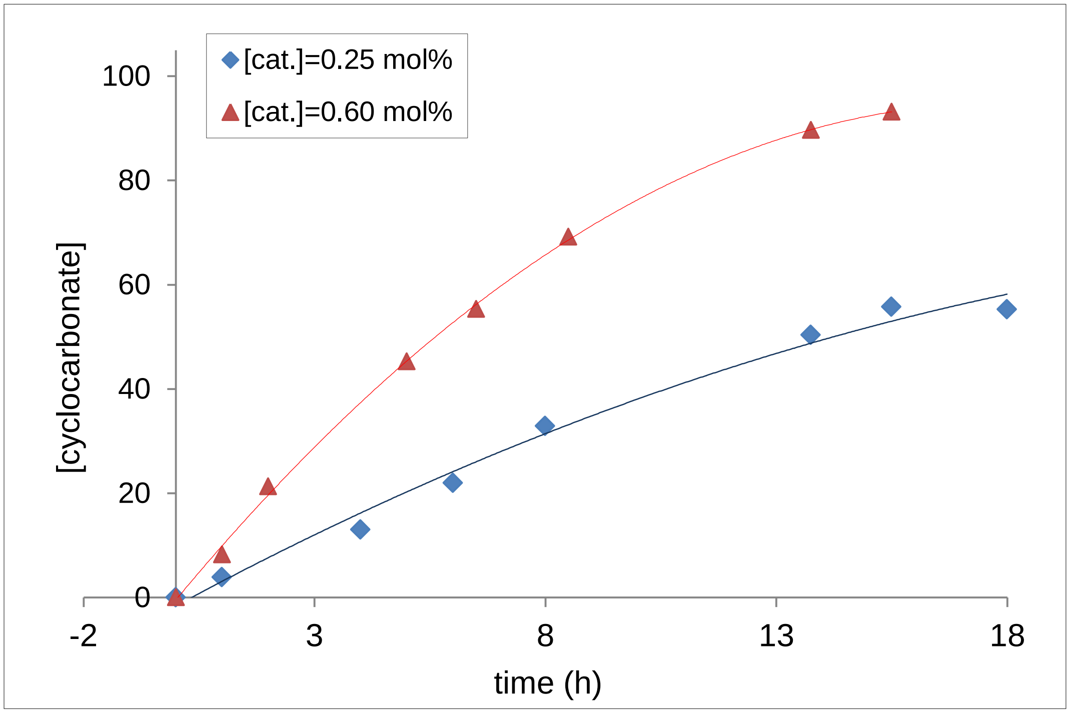

Figure S5: Cyclic carbonate formation in time at various concentrations of catalyst **1**. Conditions: 1,2-epoxyhexane (10 mmol) as substrate, 30ºC, 1 MPa of CO_2, [Zn] = [NBu_4I] = 0.25 and 0.60 mol%.

It can be reasonably assumed that at both concentrations of catalyst a steady-state domain is apparent in the first two hours.

Figure S6:

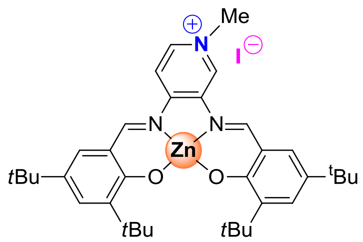

2

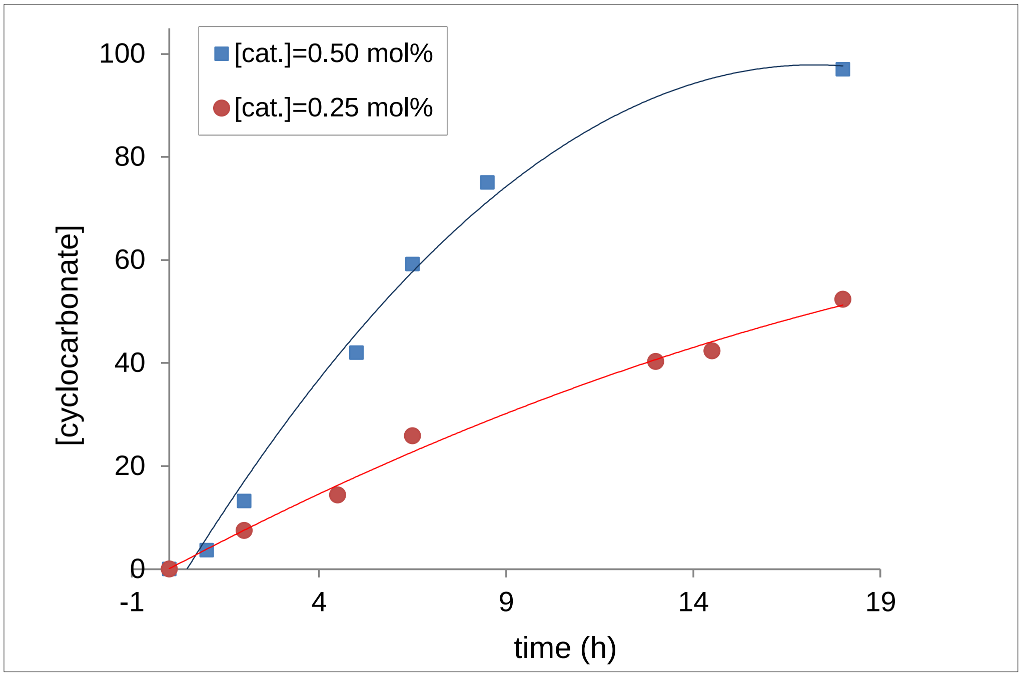

Figure S6: Cyclic carbonate formation in time at various concentration of catalyst **2**. Conditions: 1,2-epoxyhexane (10 mmol) as substrate, 80ºC, 1 MPa of CO_2, [ZnI] = 0.25 and 0.50 mol%.

It can be reasonably assumed that at both concentrations of catalyst a steady-state domain is apparent in the first two hours.
